# Erythropoietin Combined with Liposomal Amphotericin B Improves Outcome during Disseminated Aspergillosis in Mice

**DOI:** 10.3389/fimmu.2014.00502

**Published:** 2014-10-14

**Authors:** Nathalie Rousseau, Stephane Picot, Anne-Lise Bienvenu

**Affiliations:** ^1^Institut de Parasitologie et Mycologie Médicale, Hospices Civils de Lyon, Lyon, France; ^2^Malaria Research Unit, ICBMS, CNRS UMR 5246, University Lyon 1, Lyon, France

**Keywords:** invasive aspergillosis, adjuvant therapy, erythropoietin, amphotericin B, mortality, murine model

## Abstract

Disseminated aspergillosis is responsible for a high mortality rate, despite the use of antifungal drugs. Adjuvant therapies are urgently needed to improve the outcome. The aim of this study was to demonstrate that the cytoprotective effect of erythropoietin (EPO) combined with amphotericin B can reduce the mortality rate in a murine model of disseminated aspergillosis. After infection with *Aspergillus fumigatus*, neutropenic mice were randomized to receive vehicle or 7.5 mg/kg liposomal amphotericin B (LAmB) or 7.5 mg/kg LAmB combined with 1000 IU/kg EPO (16 mice per group). *Aspergillus* galactomannan and organ cultures were performed to evaluate fungal burden at day 5. Cumulative long-term survival was analyzed at day 12 post-infection according to the Kaplan–Meier method. At day 5, fungal burden was similar between non-treated and treated groups. At day 12, mortality rates were 75, 62.5, and 31% in control group, LAmB group, and EPO/LAmB group, respectively. We observed a significant decrease in mortality using EPO/LAmB combination compared to control group (*p* < 0.01). LAmB single treatment did not improve the survival rate compared to control group (*p* = 0.155). Our results provide the first evidence that EPO improved the outcome of mice presenting with disseminated aspergillosis when combined with amphotericin B.

## Introduction

*Aspergillus fumigatus* is a ubiquitous and opportunistic fungi, responsible for disseminated infections in immunocompromised patients including hematopoietic stem cell transplant, solid organ transplant recipients, and patients receiving immunosuppressive drugs (1). Invasive aspergillosis (IA) is the second most frequent invasive fungal infection in neutropenic patients (2) leading to a high mortality rate, despite the use of last generation antifungals (3). IA has a huge clinical and economical burden, and remains one of the worst fears of clinicians treating high-risk patients. While new antifungals have been developed and widely used to treat this disease, there is evidence that the outcome is highly associated with the time of treatment induction. Any delay in diagnosis and treatment decreases the rate of favorable outcome (4). Since clinical, radiological, and biological diagnoses are difficult and require time consuming investigations, it could be supposed that any additional treatment that postponed the dissemination of the fungus from the lung could help to improve the outcome. Recent research have recently focused on targeting virulence as a new paradigm for antifungals (5); virulence factors, such as proteases, phospholipases, catalases, and calcineurin, used by yeast and molds, are potential targets to improve fungal treatments (5). *A. fumigatus* growing in the respiratory tract is responsible for epithelial and endothelial cells injuries, disruption of the blood vessels integrity leading to hemorrhages and fungemia (6, 7). The translocation of the fungi from the respiratory tract to the blood is responsible for IA in immunosuppressed patients (8, 9). We wondered whether the concept of cell protective therapy in combination with antifungals may help to improve the outcome of disseminated aspergillosis for high-risk patients at an early stage of this disease. Among the potential cell protective drugs, erythropoietin (EPO) is one of the most promising drug due to its capacity to prevent programed cell death, to reduce the development of pro-inflammatory cytokine, and to enable tissue regeneration (10, 11). Recent studies demonstrated the beneficial effect of EPO or EPO analogs after acute lung injury (12) or during sepsis (13, 14). In order to document our hypothesis, we set up a murine model of disseminated aspergillosis and we studied the effect on the mortality rate of a combination of EPO high doses associated to amphotericin B.

## Materials and Methods

### Mice, fungus, and infection

Animal experiments were conducted according to the Institutional Animal Care and Use Committee guidelines of University Claude Bernard Lyon 1 (approved protocol No. BH2012-07). CBA/J female mice 6 weeks old (Janvier, France) were immunosuppressed at day 0 using intraperitoneal injection of cylophosphamide (250 mg/kg) (Baxter, France). This immunosuppressive treatment induced severe neutropenia (<0.01 G/L) from day 3 to 5. Mice were infected intraperitoneally at day 3 with 10^7^ conidia of *A. fumigatus* ATCC 13073 (LGC Standards, France). In our experiments, our objectives were not to reproduce the way of infection, but to measure the effect of a drug on the invasiveness of *Aspergillus* at an early stage of the disease. Intraperitoneal infection is a simple way of infection allowing the dissemination of *Aspergillus*, as described for other pathogens, including *Toxoplasma gondii* (15), *Influenza A virus* (16), or *Ehrlichia muris*-like agent (17).

### Treatments

After immunosuppression and infection, mice were randomly separated in three groups (16 mice per group). Based on observation in human beings (18), we calibrated the dose of liposomal amphotericin B (LAmB) (AMBISOME^®^, Gilead, Boulogne, France) at 7.5 mg/kg/day to cure 40% of mice, a success rate similar to human treatment. EPO was used at 1000 IU/kg/day, as previously described in a murine model of cerebral malaria (19, 20) and a clinical trial in human beings (21). (Group 1 (control) received the vehicle of drugs (glucose 5%) from day 4 to 7. Group 2 (LAmB) received LAmB (7.5 mg/kg/day) from day 4 to 7. Group 3 (LAmB/EPO) received a combination of LAmB (7.5 mg/kg/day) and EPO (1000 IU/kg/day) (epoietin beta, NEORECORMON^®^, Roche, Levallois-Perret, France) from day 4 to 7. Experiments were repeated twice.

### Invasive aspergillosis

To confirm that the same fungal burden was reached in each group, hearts, lungs, livers, and brains were collected from mice dead at day 5 (Group 1: *n* = 10; Group 2: *n* = 3; Group 3: *n* = 3). Two standardized fragments of each organ were sampled. One was used for culture on Sabouraud agar at 37°C during 7 days. The second was grinded in saline and after centrifugation, Platelia *Aspergillus* galactomannan EIA (Bio-Rad, Marnes-la-Coquette, France) was performed on the supernatant.

### Statistical analysis

Cumulative long-term survival was calculated according to the Kaplan–Meier method and groups were compared with the log rank test using SPSS 20.0 (LEAD Technologies, Chicago, USA). Survival time was the dependant variable. *p*-value of <0.05 was considered significant.

## Results

### Invasive aspergillosis

Intraperitoneal infection led to a reproducible disseminated aspergillosis at day 5, as demonstrated by *Aspergillus* culture positives for all organs tested and by the galactomannan antigen index (positive threshold = 1) in extracts from hearts (9.3 ± 1.8), lungs (10.3 ± 0.1), livers (10.1 ± 0.1), and brains (4.1 ± 2.5).

At day 5, there was no difference (student *t*-test, *p* > 0.1) in fungal burden between non-treated and treated groups (galactomannan index means were 8.5 ± 1.1, 9.0 ± 0.5, and 8.4 ± 0.8 for untreated, LAmB treated, and LAmB/EPO treated mice, respectively). The galactomannan levels are also similar (student *t*-test, *p* > 0.1) between groups if organs are considered (heart: 9.3 ± 1.8, 10.3 ± 0.4, and 9.8 ± 1.3; lungs: 10.3 ± 0.1, 10.3 ± 0.3, and 10.5 ± 0.2; livers: 10.1 ± 0.1, 10.4 ± 0.3, and 10.3 ± 0.2; brains: 4.1 ± 2.5, 5.1 ± 1.1, and 3.0 ± 1.3 for untreated, LAmB treated, and LAmB/EPO treated mice groups, respectively).

### Increased survival rate when EPO is combined with AMB treatment

**Figure 1:**
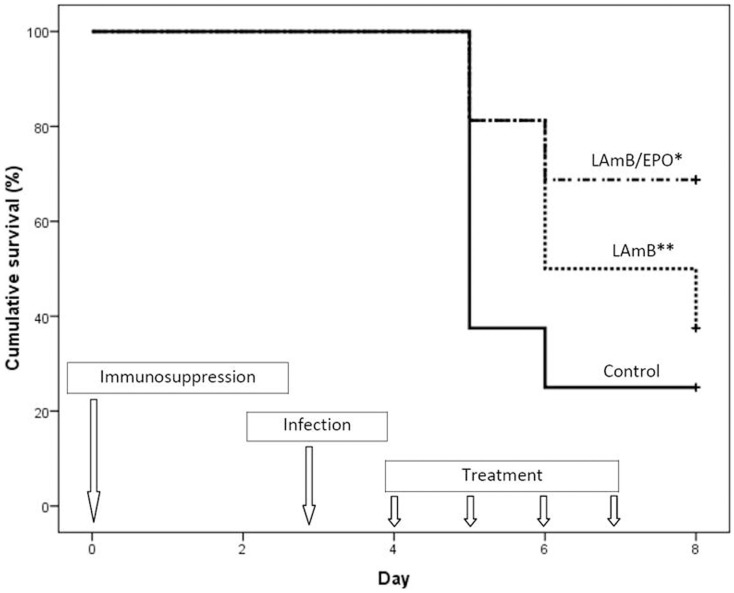
**Cumulative survival analysis of the treated and untreated mice**. Control (solid line): mice received the vehicle of drugs (glucose 5%) from day 4 to 7; LAmB (dotted line): mice received liposomal amphotericin B (7.5 mg/kg/day) from day 4 to 7; and LAmB/EPO (dash dotted line): mice received a combination of liposomal amphotericin B (7.5 mg/kg/day) and recombinant human erythropoietin (1000 IU/kg/day) from day 4 to 7; *p < 0.01 for control vs LAmB/EPO and *p* = 0.07 for LAmB vs LAmB/EPO, **p = 0.155 for control vs LAmB (Kaplan–Meier analysis, log rank test).

Mortality rates at day 12 were 75, 62.5, and 31% in control group, LAmB group, and LAmB/EPO group, respectively (Figure 1). As expected, LAmB did not significantly improve the survival rate compared to control group (*p* = 0.155), in agreement with the disease severity associated with confirmed IA. Combination of EPO to LAmB led to a slight reduction in mortality rate compared to LAmB (*p* = 0.07). More interestingly, we observed a significant decreased mortality using LAmB/EPO combination compared to control (*p* < 0.01). In LAmB/EPO treated group, *Aspergillus* dissemination was limited, as demonstrated by the reduction of fungal burden in lungs (from 33% of mice presenting infected lung in LAmB group to 0% in LAmB/EPO group). This is the first evidence for an adjuvant effect of EPO combined to LAmB during experimental disseminated aspergillosis.

## Discussion

While new antifungal drugs are widely used, disseminated aspergillosis is still associated with an unacceptable mortality rate. During neutropenia, angioinvasivness of *Aspergillus* can occur, resulting in death, despite appropriate antifungal treatment (22). The poor outcome of disseminated aspergillosis, as well as the increasing interest for EPO as a cytoprotective drug (23), paved the way for EPO combination with antifungals when a salvage therapy is required. In this situation, the antifungal commonly used as a second-line therapy for IA is LAmB (24).

Considering the need for a proof of concept study before translation to human beings, we developed a murine model of disseminated aspergillosis.

The first step was to obtain a disseminated infection after intraperitoneal inoculation of *Aspergillus* conidia, to avoid the pulmonary disease which could lead to death before dissemination. Intraperitoneal injection of pathogens with a potential to disseminate via blood route are widely used to reproduce disseminated diseases, including malaria (25), toxoplasmosis (15), erlichiosis (17), or influenza (16). Many different models of IA have been developed in the past and recently reviewed in Ref. (26). There is evidence that none of the animal model perfectly reproduces the human disease (26). The intraperitoneal infection of mice by *Aspergillus* has the advantage to lead to a hematogenous spread and to avoid the lung tropism of *Aspergillus*. After immunosuppression using cyclophosphamide, we observed a dissemination of *A. fumigatus* in heart, lung, liver, and brain, demonstrating the systemic involvement leading to a high mortality rate of 75% 5 days after infection. While limitations are obvious, our model of immunosuppressed CBA/J mice infected via intraperitoneal route is adapted to the study of *Aspergillus* dissemination.

We calibrated the infection in order to obtain a mortality rate of 75% at day 8 and the dose of LAmB to reduce mortality rate to approximately 60%, mimicking the human situation. This high mortality rate, despite the use of high doses of LAmB, is relevant to clinical observations in human beings (AmBiLoad trial) (18). The LAmB/EPO drug combination decreased by half the mortality rate (31%), demonstrating a significant effect on survival compared to control (*p* < 0.01).

Few years ago, antioxidative property of *N*-acetyl-cystein was used in mice, in association with amphotericin B, to reduce oxidative stress after intratechal infection by *Aspergillus* (27). This study showed a positive effect of the combined treatment at the pulmonary tissue level, but the effect on mice survival was not showed. To our knowledge, we report the first evidence of a successful combination of a cytoprotective drug with an antifungal during disseminated aspergillosis.

*Aspergillus* hyphae damage endothelial cells leading to disruption of endothelial cell monolayer and dissemination of the fungus. Interestingly, when EPO was combined to LAmB, the percentage of disseminated infection was reduced from 33% in LAmB treated mice compared to 0% in LAmB/EPO treated mice. This could be related to the ability of EPO to reduce endothelial permeability as recently demonstrated during intracerebral hemorrhages (28) and acute lung injury (12).

This hypothesis needs to be confirmed by histopathological studies. Further studies are also needed to confirm the safety of short-term regimen of EPO high doses. EPO is suspected to be responsible for two major side effects: tumor progression and thromboembolic events (29). The 8 weeks treatment with epoietin beta (300 UI/kg three times weekly) was associated with progression of the disease in patients with head and neck carcinomas (30). In patients with heart disease, long-term treatments with erythropoiesis-stimulating agents were associated with thromboembolic adverse events (31). In a meta-analysis aimed at evaluating the impact of epoietin beta on tumor progression and thromboembolic events, the authors concluded that these side effects were observed only if baseline hemoglobin values were increased (29). Since we used 1000 IU/kg/day EPO during 4 days, these side effects would be avoided as demonstrated by Ehrenreich et al. (32). In this safety study, patients received after ischemic stroke 3 days of intravenous high doses epoietin beta (33,000 IU/day). The hematocrit, hemoglobin, and red blood cell counts remained stable throughout the 30-day observation period (32).

In our hands, we have demonstrated the favorable effect of EPO on outcome during murine disseminated aspergillosis. Considering the low risk of side effects using short-term EPO treatment, clinical trials are urgently needed.

## Author Contributions

Nathalie Rousseau designed the experiments, carried out animal studies, and drafted the manuscript. Stephane Picot designed the experiments, analyzed the data, and drafted the manuscript. Anne-Lise Bienvenu promoted the idea, designed the experiments, analyzed the data, and drafted the manuscript. All authors critically revised the manuscript and approved the final version for publication.

## Conflict of Interest Statement

The authors declare that the research was conducted in the absence of any commercial or financial relationships that could be construed as a potential conflict of interest.
